# Cardiac Myxoma With Gamna-Gandy Bodies in a Nicaraguan Patient: Leveraging Advanced Imaging to Reduce Mortality Risk

**DOI:** 10.7759/cureus.81041

**Published:** 2025-03-23

**Authors:** Alvaro Morales, Cesar Baltodano Dangla, Christopher Romero Ríos, Ariadna Rodríguez Lezama, José Luis Huertas, Tania M Gamez

**Affiliations:** 1 Cardiology, Hospital Militar Escuela "Dr. Alejandro Dávila Bolaños", Managua, NIC; 2 Research, Hospital Militar Escuela "Dr. Alejandro Dávila Bolaños", Managua, NIC

**Keywords:** diastolic murmur, gamna-gandy bodies, left atrial myxoma, low- and middle-income country, surgical resection

## Abstract

Myxomas are the most frequent primary cardiac neoplasms, presenting with diverse clinical manifestations depending on their size and location due to obstructive effects on blood flow. In patients with pre-existing structural heart disease, their nonspecific symptoms can closely mimic acute heart failure, valvular disease, or even pulmonary thromboembolism, leading to significant diagnostic challenges. We describe the case of a 59-year-old Hispanic male patient with hypertensive heart disease with preserved ejection fraction and paroxysmal atrial fibrillation, poorly adherent to treatment. He initially presented to a primary care facility with progressive exertional dyspnea, raising suspicion of pulmonary embolism and prompting referral to our tertiary center. However, transthoracic and transesophageal echocardiography, computed tomography, and cardiac magnetic resonance imaging revealed a left atrial mass, establishing a presumptive diagnosis of a cardiac myxoma and excluding alternative pathologies. Histopathological analysis confirmed the diagnosis and revealed the presence of Gamna-Gandy bodies within the myxoma, characterized by hemosiderin deposition, fibrosis, and calcifications indicative of chronic microhemorrhages. These findings suggest prolonged vascular congestion and recurrent hemorrhages, potentially increasing the tumor’s friability and embolic risk. Without surgical resection, the patient would have faced a high risk of systemic embolism, including stroke, acute limb ischemia, or mesenteric infarction, as well as potential sudden cardiac death due to mitral valve obstruction. Early identification of the myxoma facilitated timely surgery, which was successfully performed, preventing these life-threatening complications. This case highlights the critical role of advanced imaging in differentiating cardiac myxomas from other conditions, enabling prompt surgical intervention and improving prognosis, particularly in resource-limited settings.

## Introduction

Primary cardiac tumors are rare, with an estimated incidence of approximately 0.02% [1]. In contrast, metastatic involvement of the heart is significantly more common, occurring in up to 20% of patients who succumb to cancer [2]. Among primary cardiac tumors, myxomas predominate, accounting for 50% to 60% of cases in adults and approximately 15% in children [1]. The left atrium is the most frequent site of myxomas, followed less commonly by the right atrium and ventricles. Clinical manifestations vary by location but may include dyspnea, fatigue, and, in some instances, severe complications such as heart failure or systemic thromboembolism. The presentation of atrial myxomas depends on their position and characteristics, with dyspnea and fatigue being the most frequent symptoms. However, serious complications like heart failure or systemic emboli can occur [3].

This report presents a 59-year-old Hispanic male patient with a history of hypertension and paroxysmal atrial fibrillation who developed progressively worsening dyspnea, initially on moderate exertion and later at rest. Echocardiography identified a mass in the left atrium, prompting further evaluation with cardiac magnetic resonance imaging (CMR). CMR proved crucial in elucidating the characteristics and extent of the myxoma, offering additional diagnostic value through tissue characterization, evaluation of mass mobility, and determination of the attachment site. The definitive diagnosis was confirmed through histopathology, which notably revealed Gamna-Gandy bodies. These siderotic nodules, indicative of chronic hemorrhagic changes or microhemorrhage-related siderotic deposits, are uncommon but significant findings that can provide insights into the chronicity and embolic potential of the tumor [4]. While the patient’s clinical presentation initially suggested other diagnoses, including heart failure decompensation and pulmonary embolism, the identification of the myxoma was critical for guiding timely surgical resection. Early diagnosis and intervention led to a favorable recovery, with resolution of symptoms and positive outcomes at three-month follow-up.

## Case presentation

A 59-year-old Hispanic man was referred to the emergency department from a primary care unit with a two-week history of dyspnea on minimal exertion, escalating to dyspnea at rest over the preceding three days, accompanied by intolerance to the supine position. His medical history included hypertensive heart disease with a left ventricular ejection fraction of 54% and paroxysmal atrial fibrillation, managed with carvedilol, furosemide, sacubitril/valsartan, dapagliflozin, and spironolactone, though with poor treatment adherence. He reported a 20-year smoking history but denied alcohol or drug use. 

On arrival, vital signs were stable: blood pressure 120/74 mmHg, heart rate 75 beats per minute, respiratory rate 21 breaths per minute, temperature 36.3°C, and oxygen saturation 97%. Anthropometric data included weight 60 kg, height 167 cm, and body mass index (BMI) 21.5 kg/m². Cardiovascular examination revealed a notable grade III/VI diastolic “plop” murmur at the mitral focus, with no other significant findings. Laboratory results were normal, including complete blood count, renal and thyroid function tests, procalcitonin, and C-reactive protein. Serum electrolytes (sodium, potassium, calcium, phosphorus, and magnesium) were within reference ranges (see Table 1). 

**Table 1:** Laboratory Tests Performed in the Emergency Room and Reference Parameters MCV, mean corpuscular volume; MCH, mean corpuscular hemoglobin; MCHC, mean corpuscular hemoglobin concentration; RDW-SD, red cell distribution width standard deviation; RDW-CV, red cell distribution width coefficient of variation; MPV, mean platelet volume; ECLIA, electrochemiluminescence immunoassay; TSH, thyroid-stimulating hormone.

Parameter to be evaluated	Patient values	Normal Values
Complete Blood Count		
Hematocrit	49 %	39 - 50%
Hemoglobin	15.3 g/dL	13 - 17 g/dL
White Blood Cell Count	9.72 x 10³/uL	5-10 x 10³/uL
Red Blood Cell Count	5.9 x 10^6^/µL	4-6.3 x 10^6^/µL
VCM	86.1 fL	72-96 fL
HCM	27 pg	27-32 pg
CHCM	34.4 g/dL	32-37 g/dL
Platelets	240 x 10^3^/uL	150 – 500 x 10^3^/uL
RDW-SD	41.8 %	37.2-54%
RDW-CV	13.6 %	11-16%
MPV	10.9 %	9-13%
Neutrophils	56.2 %	55-65%
Lymphocytes	29.8 %	25-35%
Monocytes	9.1 %	3-10%
Eosinophils	4.3 %	0.5-4%
Basophils	0.6%	0-2%
Neutrophils (Count)	6.1 x 10^3^/uL	5.5-6.5 x 10^3^/uL
Lymphocyte (Count)	3.22 x 10^3^/uL	20-45 x 10^3^/uL
Monocyte (Count)	0.98 x 10^3^/uL	0-8 x 10^3^/uL
Eosinophils (Count)	0.46 x 10^3^/uL	3-5 x 10^3^/uL
Basophils (Count)	0,06 x 10^3^/uL	0-2.5 x 10^3^/uL
Blood Chemistry		
C-reactive protein	0.06 mg/dL	0-0.5 mg/dL
Immunology		
Procalcitonin ECLIA	0.02 ng/mL	> 0.5 High risk of sepsis
Electrolytes		
Potassium	4 mmol/L	3.5-5.1 mmol/L
Sodium	136.6 mmol/L	136-145 mmol/L
Serum Calcium	9.2 mg/dL	8.4-9.6 mg/dL
Blood Urea Nitrogen	16.9 mg/dL	7.8-21.4 mg/dL
Total Urea	36.3 mg/dL	16.7-45.9 mg/dL
Creatinine	1.03 mg/dL	0.7-1.2 mg/dL
Thyroid Function Test		
TSH	2.67	0.4–4.0 µIU/mL

**Figure 1:**
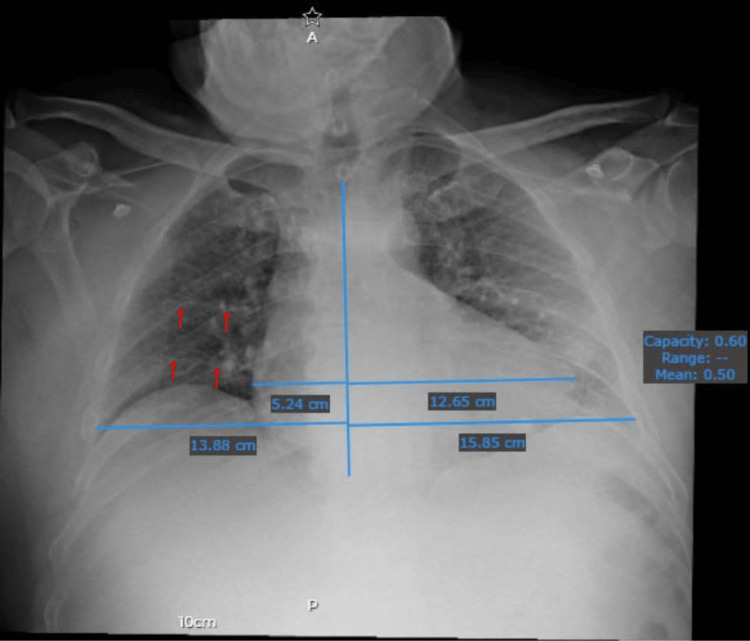
Anteroposterior Chest Radiograph The image reveals a pattern of bilateral alveolar infiltrates, most prominent in the perihilar regions, suggestive of a "butterfly wing" distribution. Linear opacities, identified as Kerley B lines (marked with red arrows), are observed in the lower lung fields, consistent with interlobular septal thickening. The cardiothoracic ratio was assessed using heart and chest widths, as indicated by blue lines: heart width, 17.89 cm; chest width, 29.73 cm. The calculated cardiothoracic ratio (CTR) is approximately 0.60 (17.89 cm / 29.73 cm), exceeding the threshold of 0.50, indicative of cardiomegaly.

**Figure 2:**
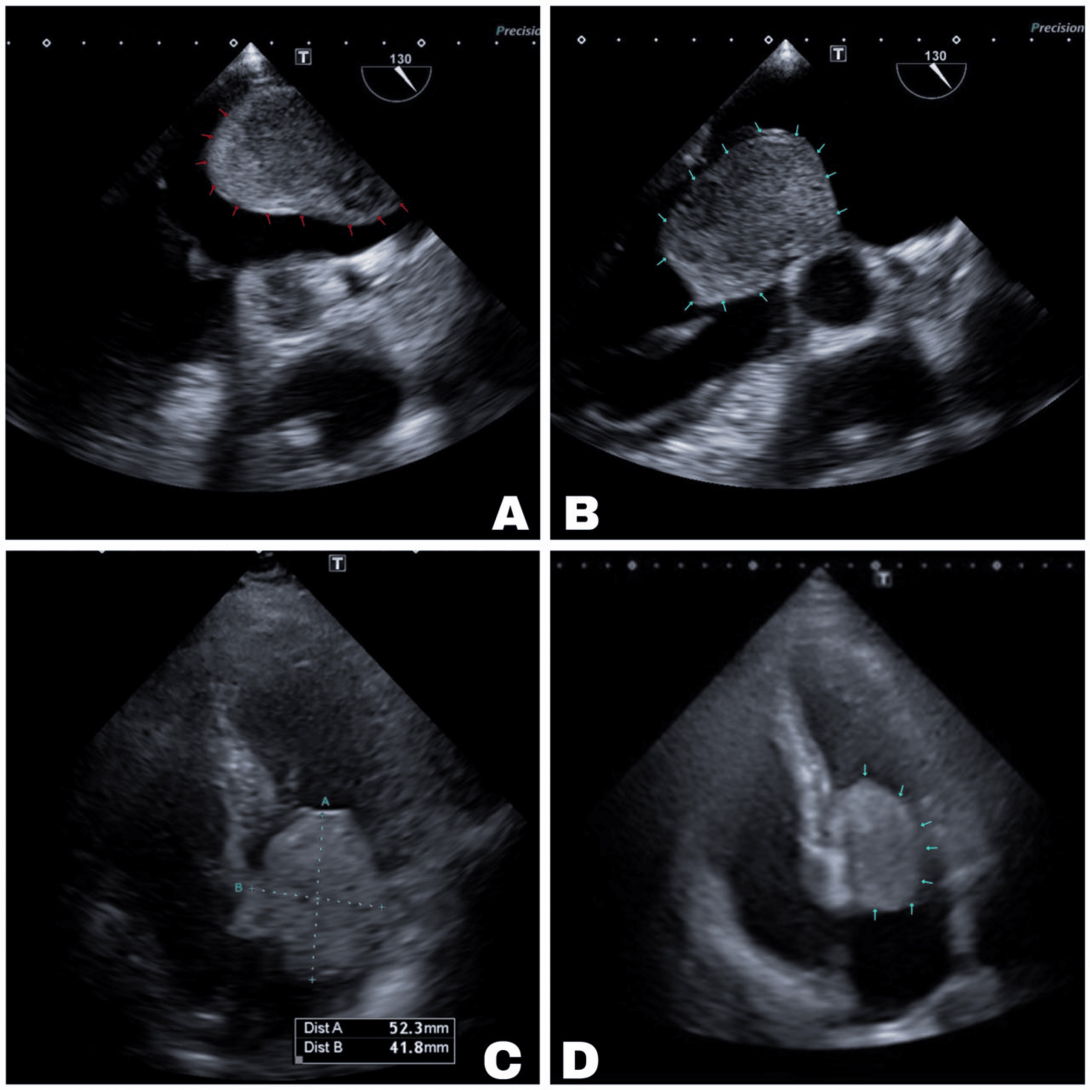
Echocardiographic Findings of an Intra-Atrial Mass A. Transesophageal echocardiogram, a parasternal long-axis view at 140 degrees, demonstrates an echogenic mass occupying the left atrium, with its outline highlighted by red arrows. B. Transesophageal echocardiogram, a parasternal long-axis view at 130 degrees, reveals an echogenic mass prolapsing through the mitral valve into the left ventricle, with its silhouette delineated by light blue arrows. C. Transthoracic echocardiogram, apical four-chamber view at the mitral valve level, shows an echogenic mass measuring 52.3 mm x 41.8 mm. D. Transthoracic echocardiogram, apical four-chamber view, depicts an echogenic mass prolapsing through the mitral valve into the left ventricle, with its outline marked by light blue arrows.

Chest radiography demonstrated patterns suggestive of pulmonary congestion and an enlarged cardiac silhouette with a cardiothoracic ratio indicative of cardiomegaly (see Figure 1). Transthoracic and transesophageal echocardiography revealed a 52.3 x 41.8 mm left atrial mass prolapsing into the left ventricle, intermittently obstructing the mitral valve orifice (see Figures 2A-2D), effectively ruling out the initial suspicion of pulmonary thromboembolism. The patient was admitted to the intensive care unit for cardiac and respiratory monitoring and initiated on enoxaparin 4,000 units. 

**Figure 3:**
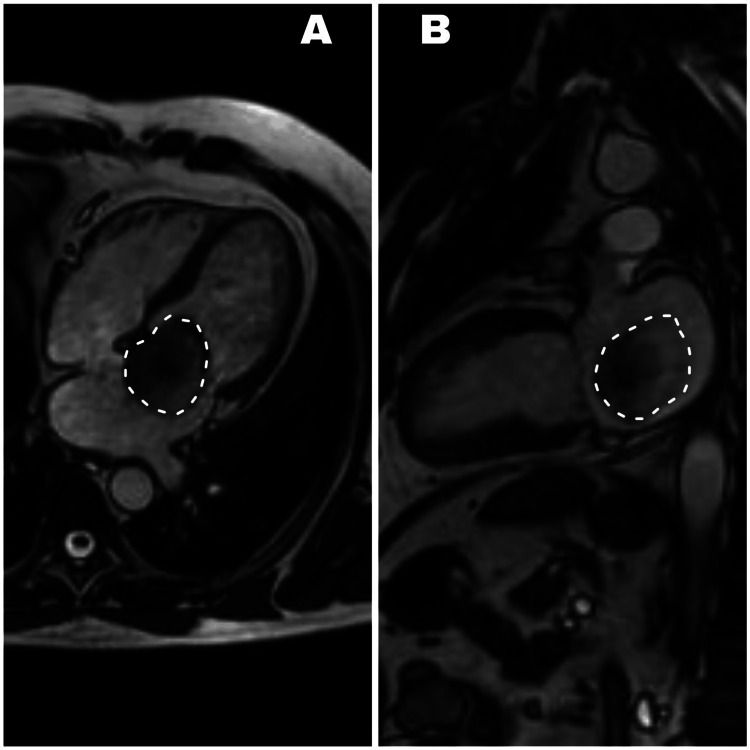
Cardiac Magnetic Resonance Imaging A mobile, oval-shaped mass measuring 55 x 39 mm, adherent to the interatrial septum, is identified. A. SSFP sequence, four-chamber view during diastole, demonstrates a left intra-atrial space-occupying lesion with apical displacement toward the left ventricular inflow tract, resulting in partial obstruction. B. SSFP sequence, two-chamber view during diastole, reveals a left intra-atrial space-occupying lesion. SSFP, steady-state free precession.

Surgery was the only viable treatment option locally. Preoperative CMR confirmed a 55 x 39 mm oval lesion in the left atrium, attached to the interatrial septum, displacing apically during diastole and partially obstructing the left ventricular inflow tract (see Figure 3). Whole-body computed tomography (CT) identified the known intracardiac mass without evidence of secondary deposits, excluding Carney complex as a differential diagnosis. Percutaneous coronary angiography showed no significant coronary artery lesions. 

**Figure 4:**
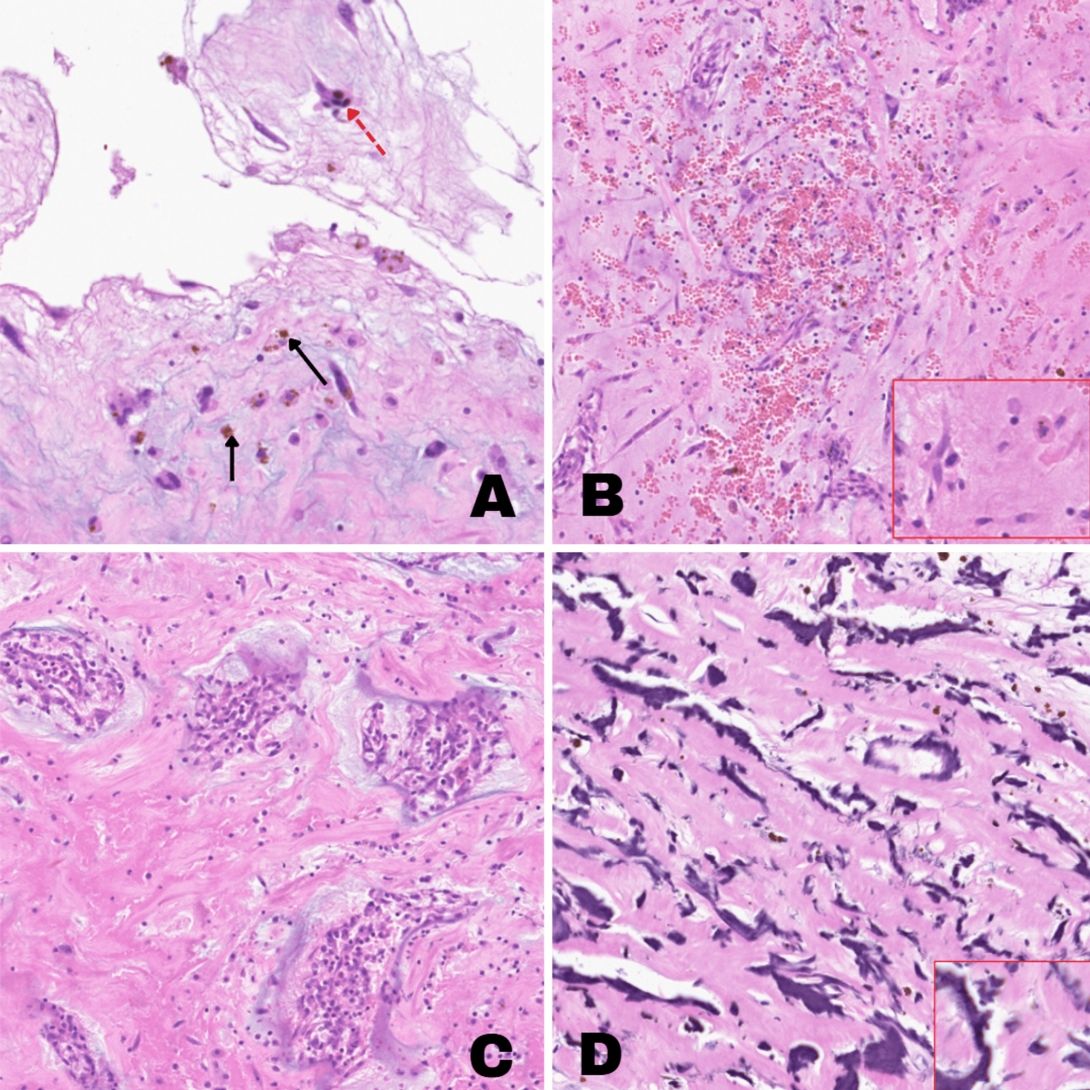
Histopathological Findings of a Cardiac Myxoma Specimen (H&E Staining) A. At 4× magnification, a stellate cell (indicated by a dotted red arrow) is observed within a myxoid matrix. Spindle-shaped nuclei with abundant eosinophilic cytoplasm and hemosiderin deposits (marked by black arrows) are prominent. B. At 20× magnification, the myxoid matrix contains notable stellate cells (outlined in red in the lower right corner) alongside areas of recent hemorrhage. C. At 20× magnification, myxoma cells are arranged in nests and cords within a fibromyxoid matrix. D. At 40× magnification, Gamna-Gandy bodies (outlined in red in the lower right corner) are identified within the cardiac myxoma, representing sclerosiderotic lesions composed of collagen fibers impregnated with calcium and iron deposits. H&E, hematoxylin and eosin

The patient underwent median sternotomy with successful resection of the left atrial tumor and its pedicle, involving the interatrial septal wall. The specimen, sent to pathology, was a polypoid mass measuring 7 x 5.5 x 2.5 cm, yellowish-brown with hemorrhagic red and gelatinous areas. Microscopic analysis revealed a myxoid matrix with stellate cells (see Figures 4A, 4B), recent hemorrhage, cellular nests and cords (see Figure 4C), and Gamna-Gandy bodies (see Figure 4D), confirming a myxoma diagnosis. 

The postoperative course was uneventful, with the patient experiencing a smooth recovery and reporting progressive clinical improvement. He was discharged after 72 hours on a regimen of apixaban 5 mg twice daily, bisoprolol 5 mg twice daily, acetylsalicylic acid 100 mg daily, spironolactone 25 mg daily, and dapagliflozin 10 mg daily.

During follow-up visits at 15 days and three months post-surgery, he remained asymptomatic, with no evidence of cardiac dysfunction or structural abnormalities on transthoracic echocardiography. Upon readmission, the patient reported a noticeable improvement in his quality of life, being able to perform daily activities with greater ease. Previously, walking 200 meters would cause significant fatigue and difficulty, whereas now he experiences only mild exertion, reflecting a meaningful enhancement in functional capacity and overall well-being.

## Discussion

Cardiac myxomas are rare benign tumors, accounting for approximately 50-60% of primary cardiac tumors in adults and 15% in children. The left atrium is the most common location, representing around 75% of cases [3]. Despite their relatively low prevalence of 0.03% in the general population, they remain the most frequent primary cardiac tumor, with an estimated incidence of 0.5 to 1 case per million people annually [4]. Myxomas can occur at any age but predominantly affect adults between 40 and 60 years, with a higher prevalence in women [5].

The precise origin of cardiac myxomas remains uncertain. They are believed to arise from residual subendocardial or multipotential mesenchymal cells within the fossa ovalis. Studies have implicated recurrent somatic mutations in PRKAR1A, as well as alterations in cAMP pathway genes such as PRKACA and PDE11A, suggesting a shared pathogenic mechanism between sporadic and familial myxomas [6]. Carney complex should be suspected in younger patients, those with multiple myxomas, or in cases associated with cutaneous pigmentation or endocrine abnormalities. In such scenarios, genetic counseling and testing for PRKAR1A mutations are warranted [6].

Cardiac myxomas manifest through obstructive, embolic, or constitutional symptoms. Left atrial myxomas frequently mimic mitral valve disease, causing pulmonary congestion, dyspnea, and orthopnea. Conversely, right atrial myxomas may resemble tricuspid stenosis, leading to systemic venous congestion. Our patient presented with progressive exertional dyspnea, ultimately worsening to dyspnea at rest. The presence of respiratory discomfort in the supine position reinforced the suspicion of intracardiac obstruction. Echocardiography confirmed a left atrial mass, with subsequent CT and MRI providing further characterization of tumor attachment and vascularity, which are critical for preoperative planning [7].

A key differential diagnosis of left atrial masses includes thrombi, other benign or malignant tumors (e.g., fibroelastoma, rhabdomyoma, lipoma, or sarcoma), and infectious lesions such as vegetations. Distinguishing features on imaging can aid differentiation; myxomas typically exhibit a pedunculated morphology, heterogeneous texture, and dynamic mobility, whereas thrombi are usually non-mobile and lack vascularization [4,8]. MRI is particularly valuable in identifying tissue composition, hemorrhagic components, and tumor infiltration, while CT can detect calcifications, which are uncommon in myxomas but may be present in older lesions [9].

Histological examination remains the gold standard for definitive diagnosis. Myxomas are characterized by an abundant myxoid stroma rich in proteoglycans, staining positively with PAS-Alcian Blue. They frequently express endothelial markers such as CD31 and CD34, consistent with their presumed mesenchymal origin. In our patient, histopathology revealed a rare yet striking finding: Gamna-Gandy siderotic nodules within the myxoma. These nodules, composed of iron- and calcium-laden degenerated collagen, are more commonly observed in the spleen, lymph nodes, and other reticuloendothelial tissues but are exceptionally rare in cardiac tumors [10-12].

The presence of Gamna-Gandy bodies within a myxoma raises intriguing mechanistic considerations. Their formation suggests chronic microhemorrhage within the tumor, potentially reflecting increased vascular fragility or prolonged blood stasis. This could contribute to a higher risk of embolization, particularly in mobile or friable tumors. Additionally, their presence might serve as a marker of tumor chronicity, indicating prolonged existence before clinical detection. Although there is no established prognostic implication, further investigation is warranted to determine whether myxomas harboring these nodules exhibit distinct clinical behavior [13].

Laboratory abnormalities in myxoma patients are often nonspecific yet may provide diagnostic clues. Systemic inflammatory markers, including anemia (49% of cases), elevated gamma globulins (45%), increased erythrocyte sedimentation rate (55%), and elevated C-reactive protein (75%), have been reported, suggesting an inflammatory response secondary to tumor presence [12,14]. In resource-limited settings, where advanced imaging may not be readily available, such abnormalities particularly in conjunction with unexplained constitutional symptoms-could raise suspicion for an underlying cardiac mass. However, they are not specific enough to differentiate myxomas from other intracardiac pathologies or systemic inflammatory conditions.

Surgical resection remains the definitive treatment for cardiac myxomas, offering an excellent prognosis with a recurrence rate of only 2.1% [14,15]. The procedure is typically performed via median sternotomy with cardiopulmonary bypass and cardioplegic arrest. In our patient, complete tumor excision was successfully achieved without complications. The overall survival rate following surgical removal is highly favorable, with a 13-year postoperative survival exceeding 95% in reported series [16]. Although recurrence is rare, long-term surveillance is essential, particularly in younger patients or those with multiple myxomas, where the possibility of Carney complex must be considered.

Postoperative complications include arrhythmias (16%), atrioventricular conduction abnormalities (26%), atrial fibrillation or flutter (35%), and, less frequently, stroke, pneumonia, cardiac tamponade, or ventricular tachycardia (2.2-4.3%) [16-18]. Early recognition and management of such complications are critical for optimizing long-term outcomes.

This case highlights a rare histopathological feature of cardiac myxoma, with the identification of Gamna-Gandy bodies adding to the uniqueness and academic interest of the report. Their presence suggests chronic intratumoral hemorrhage, potentially influencing embolic risk and tumor chronicity. The case underscores the importance of maintaining a high index of suspicion in patients presenting with unexplained dyspnea, particularly in settings where access to advanced imaging is limited.

Moving forward, improving access to CMR and enhancing multidisciplinary collaboration in resource-limited settings will be crucial for timely diagnosis and intervention. Further research into the clinical implications of siderotic nodules in myxomas may provide new insights into their pathophysiology and potential prognostic significance. From a patient-centered perspective, successful resection resulted in a marked improvement in functional status and quality of life, emphasizing the importance of early recognition and surgical management in such cases.

## Conclusions

This case highlights the diagnostic challenges of cardiac myxomas in patients with underlying structural heart disease, with the presence of Gamna-Gandy bodies as a key histopathological finding. This discovery underscores the importance of detailed tissue analysis in identifying cardiac masses and refining the differential diagnosis. High clinical suspicion is crucial in patients with progressive dyspnea of unclear etiology, especially in settings with limited access to advanced imaging. While echocardiography was useful for the initial diagnosis, CMR was essential for characterizing the mass and guiding surgical planning. A multidisciplinary approach enabled timely intervention and prevented serious complications. Despite complete symptom resolution at follow-up, continuous monitoring is required due to the risk of recurrence. This case emphasizes the importance of early intervention and access to advanced technology to improve patient outcomes.

## References

[1] Reynen K (1996). Frequency of primary tumors of the heart. Am J Cardiol.

[2] Silvestri F, Bussani R, Pavletic N, Mannone T (1997). Metastases of the heart and pericardium. G Ital Cardiol.

[3] Okongwu CC, Olaofe OO (2025). Cardiac myxoma: a comprehensive review. J Cardiothorac Surg.

[4] Kawano H, Sueyoshi N, Kawai S, Shirai T, Okada R (1993). The Gamna-Gandy body in cardiac myxoma. Cardiovasc Pathol.

[5] Grebenc ML, Rosado de Christenson ML, Burke AP, Green CE, Galvin JR (2000). Primary cardiac and pericardial neoplasms: radiologic-pathologic correlation. Radiographics.

[6] Maleszewski JJ, Larsen BT, Kip NS, Castonguay MC, Edwards WD, Carney JA, Kipp BR (2014). PRKAR1A in the development of cardiac myxoma: a study of 110 cases including isolated and syndromic tumors. Am J Surg Pathol.

[7] Bussani R, Castrichini M, Restivo L (2020). Cardiac tumors: diagnosis, prognosis, and treatment. Curr Cardiol Rep.

[8] Oktaviono YH, Saputra PB, Arnindita JN (2024). Clinical characteristics and surgical outcomes of cardiac myxoma: a meta-analysis of worldwide experience. Eur J Surg Oncol.

[9] Colin GC, Gerber BL, Amzulescu M, Bogaert J (2018). Cardiac myxoma: a contemporary multimodality imaging review. Int J Cardiovasc Imaging.

[10] Abbas AK, Aster JC, Kumar VR (2020). Robbins & Cotran Pathologic Basis of Disease.

[11] Acebo E, Val-Bernal JF, Gómez-Román JJ, Revuelta JM (2003). Clinicopathologic study and DNA analysis of 37 cardiac myxomas: a 28-year experience. Chest.

[12] Griborio-Guzman AG, Aseyev OI, Shah H, Sadreddini M (2022). Cardiac myxomas: clinical presentation, diagnosis and management. Heart.

[13] Jannete S, Claudia S, David MJ (2007). Cardiac myxomas: morphological and immunohistochemical study of 50 biopsies (Article in Spanish). Gaceta Médica de Caracas.

[14] Luo C, Zhu J, Bao C, Ding F, Mei J (2019). Minimally invasive and conventional surgical treatment of primary benign cardiac tumors. J Cardiothorac Surg.

[15] Lee KS, Kim GS, Jung Y (2017). Surgical resection of cardiac myxoma-a 30-year single institutional experience. J Cardiothorac Surg.

[16] Bjessmo S, Ivert T (1997). Cardiac myxoma: 40 years' experience in 63 patients. Ann Thorac Surg.

[17] Pinede L, Duhaut P, Loire R (2001). Clinical presentation of left atrial cardiac myxoma. A series of 112 consecutive cases. Medicine (Baltimore).

[18] Kajihara N, Tanoue Y, Eto M, Tomita Y, Masuda M, Morita S (2006). Surgical experience of cardiac tumors: early and late results. Surg Today.

